# Telehealth service delivery in an Australian regional mental health service during COVID-19: a mixed methods analysis

**DOI:** 10.1186/s13033-022-00553-8

**Published:** 2022-08-19

**Authors:** Mary Lou Chatterton, Elijah Marangu, Elizabeth M. Clancy, Matthew Mackay, Eve Gu, Steve Moylan, Amy Langbein, Melissa O’Shea

**Affiliations:** 1 Public Health and Preventive Medicine, Monash University, Level 4, 553 St Kilda Road, Melbourne, VIC 3004 Australia; 2 Deakin Health Economics, Institute for Health Transformation, Deakin University, Geelong, VIC Australia; 3 Australian Nursing & Midwifery Accreditation Council, Canberra, ACT Australia; 4 School of Psychology, Deakin University, Geelong, VIC Australia; 5 Drugs and Alcohol Service, Barwon Health, Mental Health, Geelong, VIC Australia; 6 School of Medicine, Deakin University, Geelong, VIC Australia

**Keywords:** Telehealth, Mixed methods, COVID-19, Mental health systems

## Abstract

**Background:**

COVID-19 required mental health services to quickly switch from face-to-face service delivery to telehealth (telephone and videoconferencing). This evaluation explored implementation of a telehealth mental health response in a regional public mental health provider.

**Methods:**

A mixed methods approach, combining service use data, brief satisfaction surveys, and qualitative interviews/focus groups was undertaken. Number and types of contacts from de-identified mental health service data were compared between April–May 2020 and April–May 2019. Mental health consumers and providers completed brief online satisfaction surveys after videoconferencing sessions. Attitudes and perspectives on the implementation of telehealth were further explored by applying a descriptive qualitative framework to the analysis of interview and focus group data supplied by consumers and providers. Template thematic analysis was used to elucidate key themes relating to the barriers and enablers of telehealth uptake and future implementation recommendations.

**Results:**

Total contacts decreased by 13% from 2019 to 2020. Face-to-face contacts decreased from 55% of total in 2019 to 24% in 2020. In 2019, 45% of contacts were by telephone, increasing to 70% in 2020. Only four videoconferencing contacts were made in 2019; increasing to 886 in 2020. Consumer surveys (n = 26) rated videoconferencing as good or excellent for technical quality (92%), overall experience (86%), and satisfaction with personal comfort (82%). Provider surveys (n = 88) rated technical quality as good or excellent (68%) and 86% could achieve assessment/treatment goals with videoconferencing. Provider focus groups/interviews (n = 32) identified that videoconferencing was well-suited to some clinical tasks. Consumers interviewed (n = 6) endorsed the ongoing availability of telehealth within a blended approach to service delivery. Both groups reflected on videoconferencing limitations due to infrastructure (laptops, phones, internet access), cumbersome platform and privacy concerns, with many reverting to telephone use.

**Conclusions:**

While videoconferencing increased, technical and other issues led to telephone being the preferred contact method. Satisfaction surveys indicated improvement opportunities in videoconferencing. Investment in user-friendly platforms, telehealth infrastructure and organisational guidelines are needed for successful integration of videoconferencing in public mental health systems.

**Supplementary Information:**

The online version contains supplementary material available at 10.1186/s13033-022-00553-8.

## Background

Social distancing measures to suppress spread of COVID-19 led to reduced access to social and health supports, with consequent negative impacts on vulnerable people, especially those with mental illness [1]. This led to a rapid adoption of telehealth internationally, following the successful uptake of telehealth during previous outbreaks of Severe Acute Respiratory Syndrome (SARS) and Middle Eastern Respiratory Syndrome (MERS). Telehealth is defined by the World Health Organization (WHO) as “the delivery of health care services, where distance is a critical factor, by all health care professionals using information and communication technologies for the exchange of valid information for diagnosis, treatment and prevention of disease and injuries, research and evaluation, and for the continuing education of health care providers, all in the interests of advancing the health of individuals and their communities” [2].

Fortunately, telehealth is an effective method for the delivery of health care in several clinical areas including mental health [3]. Within mental health services, telehealth has demonstrated comparable effectiveness to face-to-face care in the treatment of depression, anxiety, obsessive compulsive disorder, insomnia and reducing alcohol consumption [3]. Economic evaluations suggest telehealth interventions in mental health are cost-effective and possibly cost-saving [4, 5].

In Australia, the Commonwealth administered Medicare Benefits Schedule (MBS) approved reimbursement for medical and psychological consultations provided through telehealth as part of the COVID-19 response. State-based hospital and area mental health services also rapidly transitioned to telehealth due to COVID-19. Despite the potential value of telehealth, uptake prior to 2020 in Australia was poor due to a range of barriers affecting multiple stakeholders [6]. This is unfortunate because rural and remote communities in particular benefit from access to health services and speciality care through telehealth, reducing healthcare disparities and the shortage of health care providers [7].

Overcoming the barriers to implementing telehealth into service systems could confer significant benefit in improving access for mental health services, particularly in regional and remote areas; a key outcome sought from recent National and State based mental health enquiries [8, 9]. Recent efforts to do so have been patchy, for example, following the SARS and MERs outbreaks a Telemedicine Implementation Framework was published, but has had very low uptake globally [10].

Therefore, this evaluation aimed to explore the implementation and uptake of a novel mental health response to the COVID-19 pandemic through the partnership of a regional public mental health service with a tertiary education provider in Victoria, Australia with a view to understanding the barriers and enablers to improved uptake of telehealth in regional mental health settings. Leveraging Information Technology (IT) infrastructure and under-utilised space in the university, the regional mental health service was able to adaptively develop capability for telehealth as part of its service offerings. The following research questions were addressed:

1. What is the impact of introducing telehealth capability on service utilisation patterns in a regional public mental health service?
2. What are the patterns of telehealth use across discipline groups?
3. What is the acceptability to service providers and consumers of a telehealth model of mental health service delivery?
4. What are the enablers and barriers to uptake of telehealth consultations by mental health staff as part of an existing model of care?

## Methods

This evaluation was conducted within Barwon Health Mental Health Drugs and Alcohol services, a state funded Area Mental Health Service in the Barwon Region Victoria, Australia serving approximately 325,000 population and over 5000 clients annually. It adopted a mixed methods approach, combining quantitative analysis of de-identified service use data, satisfaction based on individual surveys, and in-depth qualitative interviews and focus groups. Consumer and mental health service staff (provider) perspectives were included in both quantitative and qualitative approaches, alongside stakeholder input from qualitative interviews. The project was approved by the Barwon Health Human Research Ethics Committee (Reference 20/103) before data collection commenced.

### Quantitative data

#### Service use

Individual records of mental health service contacts from January 1, 2019 through August 30, 2020 were de-identified prior to analysis [11]. The service records were administrative data reported to the state of Victoria that commissions services, and contained the number and length of contacts with consumers but did not contain clinical assessments or therapeutic interventions. Service records contained all contacts including brief contacts by telephone or asynchronous contacts through mail and text. Contacts of less than 10 min were assumed to be non-therapeutic encounters and excluded from analyses. Remaining contacts were grouped to match the initial localised lockdown period that limited availability of face-to-face service contacts and when videoconferencing became available in 2020 (April 1 through 30 May) (Additional file 1: Table S1). Contact data from 2019 was matched to the same period in 2020, accounting for potential seasonal variation. Descriptive statistics were used to compare total numbers of contacts by type and across subcentres.

#### Brief surveys

Mental health consumers and providers utilising videoconferencing between September and December 2020 were invited to complete a brief survey via messages presented at the conclusion of videoconference sessions. Participants were redirected to a Qualtrics survey, commencing with a plain language statement approved by the ethics committee.

The consumer survey contained five questions related to satisfaction with the technical connection, personal comfort, and overall experience. A three-item provider survey captured perceptions of satisfaction with the technical connection and the ability to meet assessment or therapeutic goals of the session. Both surveys were adaptations of existing questionnaires [12, 13], with copies available in the additional materials (Additional file 1: S2, S3).

Descriptive statistics (number, percent) reported survey responses by response category separately for consumers and providers.

### Qualitative data

Interview data were gathered from service providers, consumers, service leaders and managers, to explore experiences of the uptake of telehealth across the service from consumers and providers.

Semi-structured interview schedules guided interviews and focus groups conducted within the qualitative arm of the study (Additional file 1: S4, S5). Consumers were offered individual interviews to preserve confidentiality whilst service providers and managers could participate either individually or as part of focus groups. Semi-structured interviews with service providers focussed on their perspectives regarding the acceptability of telehealth for the mental health services they delivered with consumers, barriers and enablers to the use of the platform, and their views regarding incorporation of telehealth as a service platform beyond the pandemic. Semi-structured interviews with service managers and leaders focussed on identifying key success factors for the implementation of telehealth in similar services. Consumer interviews explored personal perspectives of the acceptability of telehealth for their mental health care, the ease of use of telehealth, and their views on whether telehealth should be retained beyond the pandemic.

Consumers were recruited via social media platforms, emails from the health service’s consumer advisory group, waiting room flyers and a message at the end of videoconference sessions. Consumers were required to be over 16 years, and to provide informed consent (and parental consent for consumers under 18 years) and were offered a $30 gift voucher in recognition of their time. Eligible service providers included all clinical staff who had the option to utilise telehealth as part of their clinical work with consumers, including medical, nursing and allied health staff employed in community mental health teams. Service staff were advised of the study via emails and newsletters from telehealth administration staff and service management.

In all cases, prospective study participants were asked to contact the external research project manager if they were interested in participating in an interview or focus group. On contact, they were provided with a plain language description of the project and consent form. To facilitate clinical staff participation, researchers travelled to clinical practice sites during working hours and provided service providers with a plain language description of the project and consent form. Once consent was obtained, interviews with researchers experienced in qualitative research methods were scheduled, conducted and audio-recorded. Service providers, managers and leaders were not provided with any incentives but participated in interviews or focus groups during work hours. Interviews were transcribed by research team members, with identifying information removed prior to analysis and separated from participant consent material for anonymity.

Data analysis of qualitative data was guided by a descriptive qualitative framework, suitable for answering specific questions about participants experience of events, for example, their adoption of a new treatment approach [14]. Template thematic analysis was applied to all semi-structured interviews and focus group responses. Template analysis is a form of thematic analysis that utilises hierarchical coding whereby emerging themes are organised into clusters, nesting narrower themes within broader ones [15]. The approach allows for the use of a priori themes relevant to the research questions, in this case, themes related to stakeholders’ acceptance of telehealth as a mental health treatment mode, along with identified barriers and enablers of uptake [16].

## Results

### Quantitative analyses

#### Service use

The initial data set included over 200,000 service contacts from January 1, 2019 through August 30, 2020. The distribution of consumers across age groups was similar between 2019 and 2020 as shown in Additional file 1: Table S8. In total, 20,023 contacts were recorded from April to May 2019. There were 13% fewer total contacts during the same period in 2020 (17,360) which coincided with the first wave of COVID-19 in Australia and lockdown restrictions. Direct contacts (face-to-face) totalled 10,949 in April–May 2019 and decreased by 62% during April–May 2020 to 4185. Telephone contacts increased by 36% from 8969 in April–May 2019 to 12,156 in April–May 2020. Videoconference visits totalled 4 in April–May 2019 and increased by 22,000% to 886 in April–May 2020 (Table 1).

**Table 1:** Mental health service utilisation comparing April–May 2019 to 2020

Contact type	April 2019	May 2019	Total	Percent of total %	April 2020	May 2020	Total	Percent of total %	Percent change 2019–2020 %
Face to face	5045	5904	10,949	55	1904	2281	4185	24	− 62
Other synchronous	50	51	101	1	55	78	133	1	32
Telephone	4025	4944	8969	45	6252	5904	12,156	70	36
Videoconference	2	2	4	0	374	512	886	5	22,050
Total	9122	10,901	20,023	100	8585	8775	17,360	100	− 13

The peak in total number of videoconference contacts occurred in May 2020 and dropped to 350 in July 2020 (Additional file 1: Fig. S6). The total number of telephone contacts continued to increase reaching a peak in August 2020 (Additional file 1: Fig. S7).

**Fig. 1:**
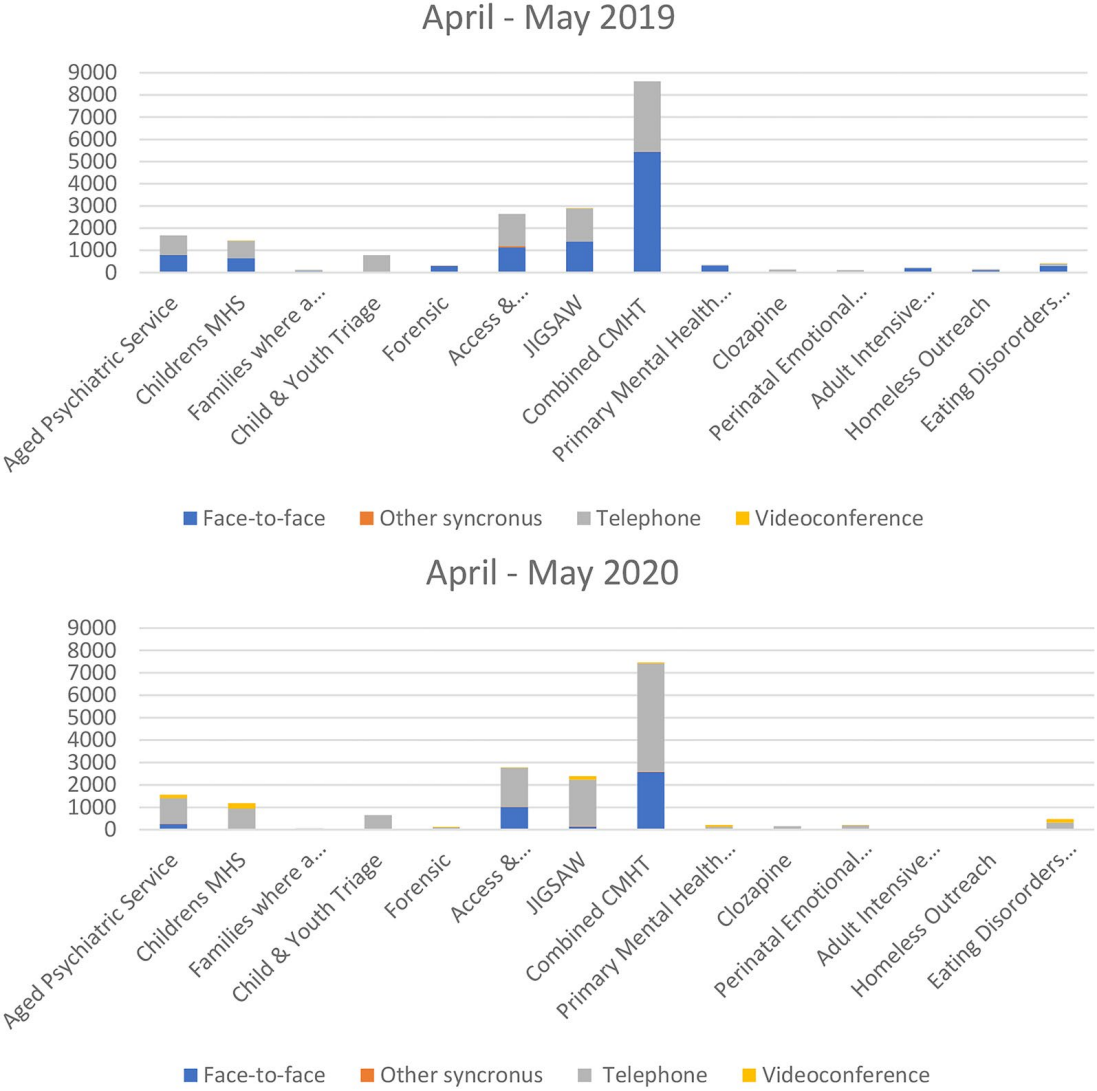
Contact type by service subcentre and year

Subcentres with the largest increase in videoconferencing use between April–May 2019 and 2020 were Child and Adolescent Mental Health Service, Jigsaw Youth Mental Health Service and Eating Disorders Service (Fig. 1, Additional file 1: Table S9).

#### Surveys

Approximately 1020 telehealth consultations were conducted between September and December 2020 following which consumers and providers were asked to complete surveys for this study.

**Fig. 2:**
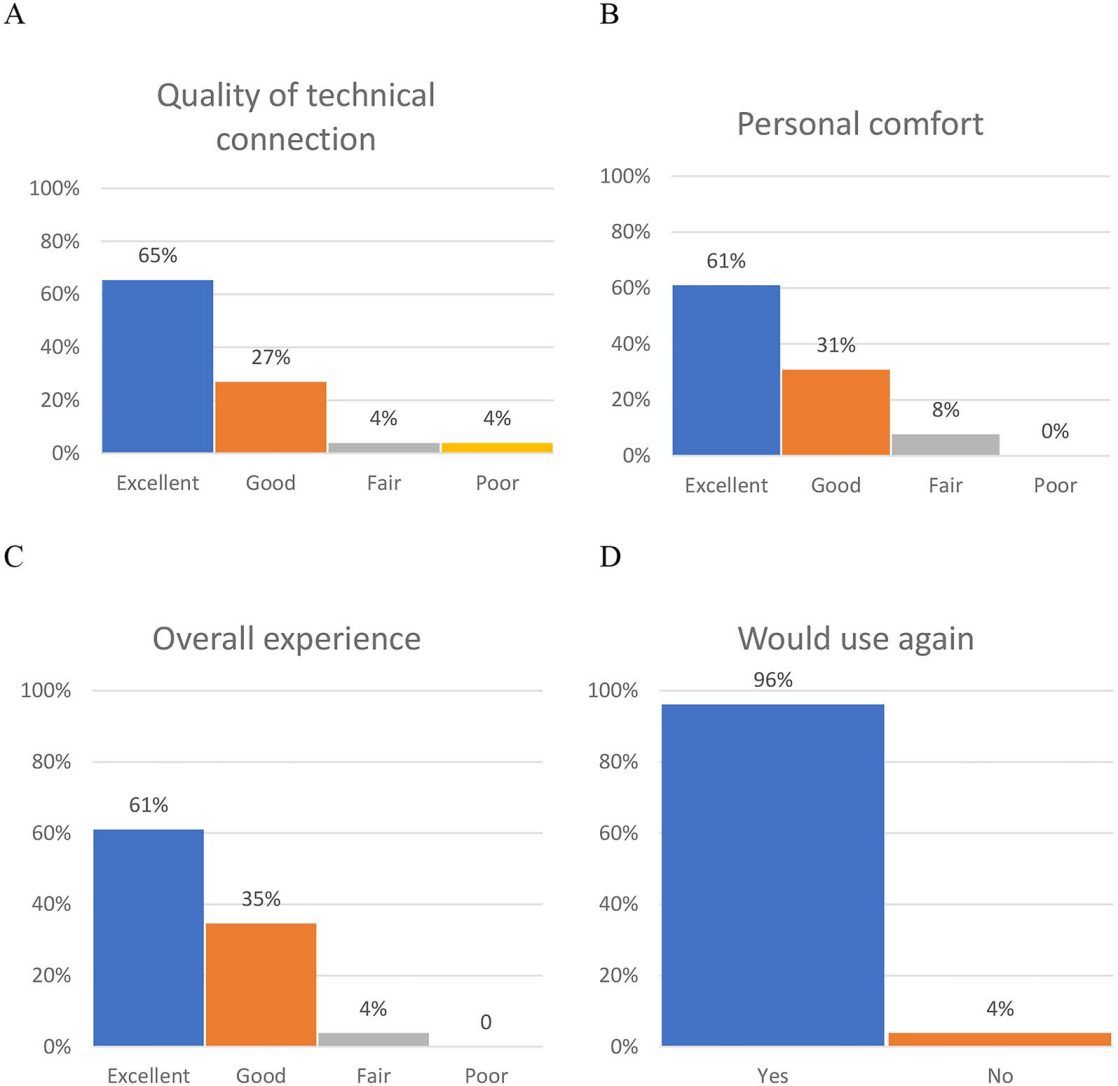
Consumers’ reported satisfaction with videoconference telehealth on **A** quality of the technical connection, **B** personal comfort, **C** the overall experience and **D** the response to ‘Would you use telehealth again?’

Forty-one consumers began the online survey by acknowledging review of the plain language statement and agreeing to participate. Of those, only 26 consumers provided responses to the questions regarding satisfaction with the telehealth session and were used in the analysis. Only one consumer (4%) reported this as a first telehealth session (Additional file 1: Table S10). Consumers rated the following attributes as good or excellent: quality of the technical connection (92%), overall experience (86%), satisfaction with personal comfort (82%), and 96% said they would use telehealth again (Fig. 2).

**Fig. 3:**
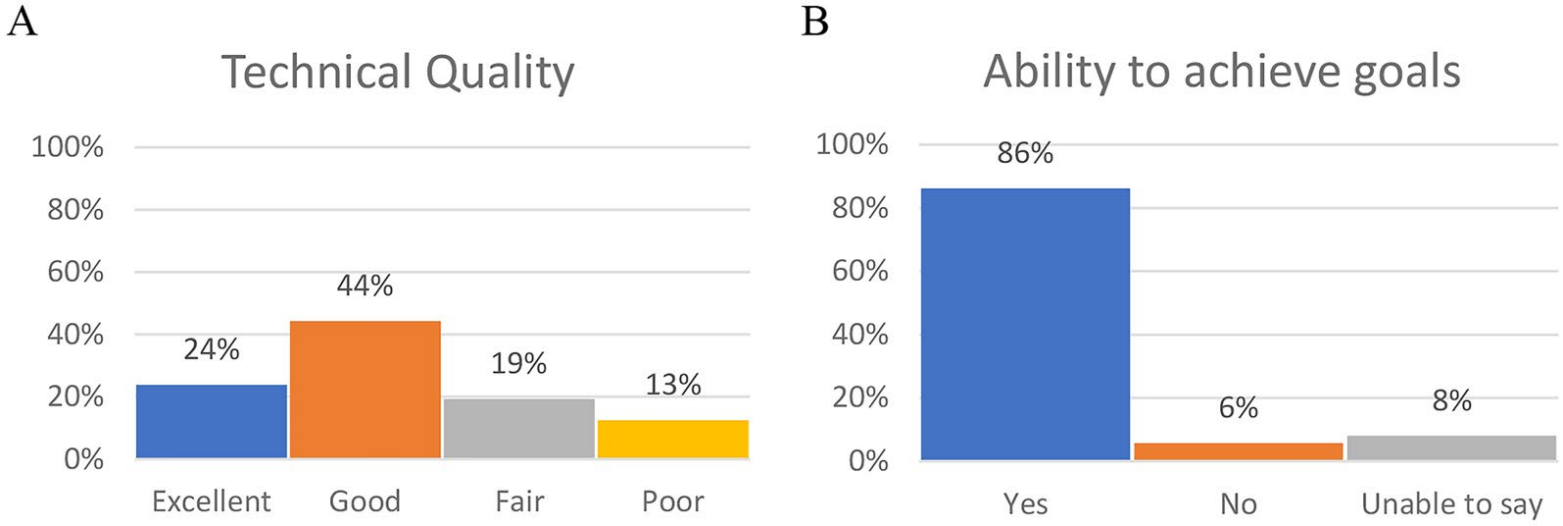
**A** Providers’ rating of videoconference telehealth on technical quality and **B** providers’ ability to achieve assessment or treatment goals in the session

For providers, 88 responses from four mental health teams were available (Additional file 1: Table S11). Providers rated quality of the technical connection as good or excellent (68%) and 86% reported they were able to achieve their assessment or treatment goals (Fig. 3).

### Qualitative analyses

#### Service providers

Thirty-two providers communicated their opinions through interview or focus groups around four overarching themes: exploring utility, addressing feasibility, working with the nuances of the telehealth service delivery, and looking toward success.

#### Exploring utility

Attitudes to the utility of telehealth varied significantly. Whilst some providers strongly valued telehealth as part of the service response during COVID-19 and potentially beyond, other providers held cautious or negative attitudes, choosing not to adopt it, or describing early failed attempts to work with the modality which led to perceptions that the modality was ineffective. A small number of providers described concerns that telehealth was being proposed as an alternative to current service models, aiming to increase cost efficiencies at the expense of client care.

Providers felt that telehealth was more suited to some service areas than others. Many providers who worked with adult (26+ years) community mental health teams or specialist drugs and alcohol services felt telehealth was poorly suited to their work demands which they described as typically opportunistic, requiring flexibility (e.g., phone, drop-in interventions). This was unavailable with telehealth which they viewed as only suitable for pre-planned clinical work. They also noted limits to telehealth for many clinical tasks such as physical examination, medication administration and crisis assessment, but noted that telehealth might be suitable for lower-risk practice areas and enhance access to medical staff consultations.

P1: “*You can’t perform a physical exam over Telehealth, you know and that is still sometimes required in mental health…you know, to assess for side effects from medication and look at their mobility*.”

In contrast, providers within teams who engaged in extended mental health assessments and focussed psychological therapies felt that telehealth closely replicated face-to-face modes for these interventions and ensured continuity of care for their clients. These providers, most often working with children and young adults (< 25 years) or facilitating psychological-focussed interventions, described telehealth as superior to phone-based care, which was an unsuitable substitute to in-person care due to challenges in establishing consumer engagement and the inherent loss of visual cues. These providers noted that most clients, including those with early reluctance, were eager to continue care in telehealth, finding it highly acceptable and convenient during a period of elevated stress. When face to face services were available, some clients returned to face-to- face, whilst others expressed a preference to continue care in telehealth, citing it as more convenient and effective.

P8: “*One of the main similarities is about kind of the interpersonal kind of relationship between myself and the client so trying to be collaborative…trying to be flexible…trying to be focused and deliver psychology intervention*.”

P24–31: “*A couple of families that were in the first few sessions were complaining about telehealth and why they couldn't come in and then we finally got back into the office I rang and said ‘good news, we're back in the office’ and they went ‘oh no, we’re quite comfortable using telehealth now’*.”

Regardless of the team in which providers worked, most spoke highly of the value of telehealth to promote equity of access beyond the pandemic, particularly for rural clients and other cohorts who may experience barriers to treatment such as clients with disabilities, older clients and those with particular mental health conditions (e.g. agoraphobia). Several providers noted that telehealth was more acceptable to younger clients who viewed the mode as an extension of the way they were living their life in school and social arenas, but less so to older cohorts who they felt had more difficultly navigating the service mode. However, providers also spoke of success for older clients, and that achieving competency with telehealth built self-efficacy and potentially supported engagement in telehealth for other health needs.

*P8: *“*The ease of clients could join a session without having…to kind of factor in the travel time …because Barwon Health covers quite a geographically large area*”.

P8: “*We’ve got certain clients who are quite anxious about leaving the house or due to their symptoms really struggled to be around crowds so even having telehealth as part of like a graded kind of therapy of eventually getting someone to challenge themselves and build up towards attending face to face over time*”.

In parallel, many providers noted that telehealth supported the work they did in various ways. In particular, they recognised that telehealth provided flexibility, reducing travel times for clients who were amenable to telehealth service provision, potentially promoting improvements in their work efficiency. Other advantages cited included using telehealth to facilitate interprofessional practice and case conferences with multiple staff, clients and carers more efficiently and easily, as well as undertaking supervision of remote-working staff and students.

P24–31: “*we’ve just noticed it at meetings now, like if somebody's you know at a different worksite that day…without having to travel like so for care coordination and things like that…a really good option for that in terms of planning treatment with other health professionals involved*”*.*

#### Working with the nuances of the telehealth service delivery

Providers who readily adopted telehealth reflected several ways in which telehealth differed from in person clinical service. Several providers remarked that continuous use of telehealth was more fatiguing than other forms of care, which they attributed to practical issues such as eye strain as well as a perception that they needed to work ‘harder’ or ‘differently’ to create and sustain client engagement. Providers spoke to how telehealth simultaneously enhanced and limited their access to relevant clinical information about clients. Most spoke about losing access to the client’s “energy”, which was often a useful tool to understand a client’s mood state. For other providers, particularly those working with consumers suffering from eating disorders, loss of visibility to a client’s ‘body’ was particularly problematic.

P2: “*If you are sitting with someone who is really sad, you can feel the sadness or the weight or heaviness whereas through a video you can’t tune into those kind of non-visual cues.*”

P24–31: “*You only see a very small portion of that person and actually having the whole body in the room gives you very important clinical information* [patients with eating disorders] *that you just couldn't really pick up on or that they weren't able to discuss or share.*”

Some providers also spoke of the impact of losing the liminal space between the waiting room and the therapy session itself which they noted was otherwise helpful in their assessment of a client’s mental state but also acted as an important bridge between the client’s personal and therapy time. Several providers remarked that this had the potential to blur the boundaries between clinical and private spaces for clients and themselves when operating in their own personal spaces during periods of lockdown. Amongst other consequences, many providers described how this gave rise to distractions to therapeutic work, particularly for children who were more open to being side-tracked by toys, devices and other home activities (“kids just being able to walk away!”). Even for adults, providers reflected on the loss of the traditional ‘therapy space’ noting that some clients felt it appropriate to ‘multi-task’, with some clients attending sessions alongside other personal activities (“on the golf course” and in the “taxi”).

P2: “*I think for some people it became harder to set the boundary around this is a professional appointment where you need to turn up and actually really just attend to the reason that we’re here.*”

P11: “*Even like you know in the waiting room or something just like picking up on clinical information like on the person's presentation like you don't get that with telehealth.*”

Providers also remarked that telehealth provided insights not available through in-person work, such as better understandings of a client’s personal space (such as their home environment) and family connections, which enriched formulations and treatment options. Some providers also reflected that opportunities to engage in treatment at home was a positive for some clients, since familiarity and safety of the home environment supported them to be more open.

P11: “*maybe telehealth is a great first step like just to engage them so they're too scared to go into a clinic you know 'cause that's just feels too overwhelming well like this could be a great option ‘hey how about we start at home in your lounge room where you feel comfortable and safe and then we can work towards you coming into the clinic face-to-face’*.”

P2: “*Actually you can get a glimpse into someone’s home life I guess when they’re on Telehealth*”.

Discussions of the nuances of telehealth led to reflections on adjustments that service providers made to their usual clinical practices. Most noted that adjustment required trial-and-error and forward planning for managing inevitable IT disruptions, e.g. agreements to revert to telephone if internet connections failed. Many reduced session times to counter both their own and consumer “zoom” fatigue and reviewed appointment scheduling, wherever possible avoiding back-to-back telehealth or other online interactions (for example, online schooling for students). Others noted the importance of more frequent check-ins with clients and seeking clarifications to counter some clinical data lost in online interactions. Despite early scepticism, most active users of telehealth remarked that tools and worksheets from face-to-face clinical work could be successfully transposed, although adjustments were time-consuming in the early phases.

P2: “*We made a decision as a clinic to try and limit our session times on Telehealth from typically an hour, we tried to reduce them down to 45 min…I guess in recognition that it’s actually quite tiring.*”

Noting concerns about managing risk via telehealth, providers emphasised the importance of contingency planning, such as negotiating how providers should assist where a consumer reported feeling unsafe in an online consultation. Sometimes this involved engagement of other family members at home in safety planning, which for one provider represented a positive outcome of the telehealth service model as it encouraged more family-inclusive practice. Most emphasised the need to carefully establish new expectations and responsibilities in telehealth work, a task most acknowledged was a work-in-progress.

P24–31: “*If you're dealing with someone who's very volatile or potentially suicide risk telepsychiatry needs big backup, you need someone else in the house who you can call who can intervene to make sure they’re alright*.”

P8: “*You don't know why they’re not in the waiting room for the telehealth calls so then there's a whole kind of range of follow up to make sure that they're safe and ok.*”

#### Addressing feasibility

Providers identified both barriers and enablers to the effective uptake of the service mode. A prevailing theme highlighted inadequate IT infrastructure being available to enable telehealth, including a lack of suitable hardware and high-speed internet access to facilitate seamless telehealth services.

P9: “*Gathering information for perhaps like a developmental history or a parents family of origin story, we might just do that…sometimes I was choosing to do that over the phone and I guess I personally probably preferred that for those times because I had issues with Telehealth from home that were technical issues like internet speed and that kind of thing*.”

P12–23: “*…we gave up on it pretty quickly and moved just to phoning people because you would lose people*.”

Referring specifically to the period when most providers were working from home, many noted that access to confidential spaces amidst their home environment was frequently challenging. While some providers felt processes to engage service users via telehealth were easy and straightforward, many found the mode cumbersome and were not motivated to overcome these early setbacks (“*It was just one extra step*…”). Many providers reported that other video/audio platforms they were adopting in other professional or personal areas of their lives in response to COVID-19 were more user friendly and intuitive than prescribed telehealth consultation platforms, typically reverting to phone consultations when face –to-face was not possible. Others acknowledged limits to their own IT capability (“*I’m not particularly tech-savvy*”) which they understood led to reluctance to adopt telehealth.

P8: “*I'm thinking of 1 case where we actually just could not get her to connect to the platform so we were like talking through the steps, we were trying to pull up like the way she was accessing it on our own mobile devices to try and work out what was going wrong.*”

P1: “*There was a number of reviews where we had to either abandon go on a phone or abandon the audio and keep the video and use the phone, the telephone for the audio. So, it became a source of frustration and…distraction and also became unpopular with some of the doctors as well because of the problems…therefore it was underutilised and still is*.”

Provider perceptions of consumer’s attitudes to telehealth represented both enablers and barriers to uptake. Several providers felt the service mode would not be acceptable to consumers with more complex mental health concerns. Some believed that consumers would be concerned that telehealth did not adequately maintain confidentiality and privacy, particularly those with paranoid symptoms. Others referred to socio-economic factors they viewed as barriers for consumers. This included requirements for consumers to have infrastructure e.g., phones or computers and sufficient internet data which they felt consumers often did not have access to. Some providers felt uncomfortable at the need for consumers to utilise internet data for telehealth within a public health service. Notably, most acknowledged that they had not expressly sought views and preferences of consumers in deciding whether to offer telehealth.

P24–31: “*Another issue for eating disorders and specifically is a lot of patients don't like seeing themselves on the screen*.”

P12–23: “*It made me really reluctant to phone anyone because I couldn't tell them unequivocally with confidence what it was going to cost them, whether it's going to cost*.”

P12: “*I found some of them were concerned about privacy and I work with lots of clients that are coming through the criminal justice system so some of them are quite concerned about privacy…mainly it was privacy and finances that I thought was the major barrier to them accessing telehealth.*”

In contrast, other providers identified a range of factors that they viewed as supporting their use of telehealth. Providers holding generally positive attitudes towards telehealth were more likely to adopt it and problem-solve early technical challenges for themselves and clients. Early positive and successful experiences of telehealth were seen as motivating participants to continue offering telehealth as a service option. Some of these providers noted that over the period of COVID-19, they had access to improved telehealth infrastructure that supported its uptake, as well as efforts to overcome barriers such as economic ones by the provision of data cards to clients.

P8: “*We were kinda surprised by how many people were willing to give it a go and Barwon Health actually would allow us to give data cards to people who couldn't access due to not having sufficient mobile data so we were working really hard if anyone was interested that we could overcome the barriers*.”

P1: “*I think when you made it clear to people that we would handle the side of the technology that was a relief for them, that they didn’t have to do anything other than, you know, look at the screen and talk.*”

#### Looking toward success

Perhaps strikingly, providers appeared to draw on a range of contrasting decision-making frameworks when considering whether to use telehealth with their clients. This included consideration of their own service context, client presentations and leadership factors in their own team, pragmatic considerations such as available IT infrastructure, client preferences, and personal attitudes and preferences. One provider explicitly explored their discipline in consideration of appropriateness in adoption of telehealth. Observations of disparate decision-making frameworks used by providers in considering the adoption of telehealth were reflected in a shared view that the service lacked an overarching vision and/or clear guidelines for the adoption of telehealth to guide individual team and clinician decisions as to when and how to adopt telehealth locally. While some teams appeared to adopt a strong stance regarding the adoption of telehealth for the service delivery during the COVID-19 period, others described a lack of clarity that saw them revert to traditional approaches such as the telephone for the majority of their work when face-to-face consultation was not available.

P12–23: *“…being able to kind of assess what clients are more likely to want to use it or able to use it and that's more likely to lead to successful outcomes. So, individualising that care.”*

P8: “*I think from the service as a whole being you know Telehealth kind of positive and so when you're talking to a client you kind of presenting them as equal options that you know*.”

When asked about how telehealth might be more successfully adopted within the service model of the organisation, second to investment in robust, effective, stable and user-friendly platforms and infrastructure for telehealth, development of such clear guidelines was strongly advocated. Consistent with this recommendation, several providers also spoke to the need for strong leadership in telehealth implementation. They saw this as crucial to promoting known benefits of the model and addressing barriers including assertively working with pockets of resistance in the service which had the potential to undermine more positive views elsewhere. Amongst other recommendations was the development of mechanisms to share knowledge and experience, particularly from successful adopters to less successful adopters, through targeted education and training. Whilst it was acknowledged that the suddenness of the pandemic exposed a lack of planning for the adoption of telehealth, recent experiences could now be leveraged to promote preparedness and success for the future.

P10: “*Maybe there could be a bit more collaboration between the teams where it was more ideal compared to like the AIS teams*.”

P8: “*Us really promoting it I think rather than seeing it is like a substitute or like a lesser kind of form of therapy…really kind of championing it and being confident in the way we talk about it*.”

#### Consumers

Six consumers were recruited and interviewed with template analysis revealing four overarching themes.

#### Benefits of access to telehealth

All six consumers had utilised telehealth during the COVID pandemic. All bar one consumer expressed strong acceptance of telehealth as part of their mental health care presuming they could access a suitably private space in their home, reflecting that it provided a very good substitute for face-to-face care. Most consumers agreed that it was preferable to phone-based consultations which did not facilitate the same ‘connection’ or rapport with their service provider. This was contrasted by one consumer who expressed distrust of the technology and fears around it invading their home and other private living spaces. Another consumer reported discomfort in looking at their own images during video consultations. Most consumers reflected that availability of telehealth throughout the pandemic enabled them to commence or continue their mental health treatment safely and during a time of heightened stress.

C5: “*[Telehealth] was like face-to-face…just not in the same room. I felt like I got to know [the psychologist] and was able to trust him…he wasn’t a stranger because I could actually see him*.”

C4: *“I don’t feel comfortable with knowing Zoom is…identifying everything in my house. As soon as you give it access to your camera and your microphone….Privacy-wise, I don’t like it and I’ll never use it again*.”

C1: “*I found it a bit daunting at first just having to look at the image of myself,*.”

#### Utility

Telehealth was generally viewed as enhancing access to mental health treatment beyond the pandemic with most consumers willing to use it again for some, but not all aspects of their mental health treatment. For example, consumers who utilised telehealth for psychological therapies agreed that it was similar to face-to-face therapeutic encounters and believed it to be as effective. They also saw telehealth as a suitable platform for routine reviews including medication reviews. Many saw telehealth availability as particularly important for consumers who struggled with low energy and depression, as well as anxiety, for whom face-to-face attendance could be a barrier to engagement.

One consumer’s carer viewed it as less reliable for initial assessments, where they were concerned that important clinical information and cues were “lost in translation with telehealth’. This was viewed as particularly problematic for consumers suffering from eating disorders when a full examination was seen as critical to development of an effective treatment plan.

C1: “*…summoning the energy, and strength if you like, to get up and get organised to leave the house, it’s sometimes, it is near impossible sometimes… [telehealth] allowed me to access services*.”

C2: “*I think there needs to be some discussion around where and when it’s appropriate and when it’s not*.”

#### Managing telehealth

Almost all consumers reflected on factors that supported as well as disrupted their satisfaction with telehealth. As telehealth services are dependent on stable internet connectivity, unreliable and inconsistent internet connectivity was a limiting factor for the effectiveness of telehealth for most participants. Several consumers noted that the telehealth platform used by the service was not immediately easy to navigate, with some anxiety arising ahead of the first session. Many remarked that having support from the service ahead of the first telehealth session and access to troubleshooting supported their adjustment to telehealth and growing confidence with it over time. For one consumer, this built their confidence with technology in general and their willingness to engage in telehealth for other aspects of their healthcare. Nevertheless, all agreed that presence of a stable platform and reliable internet was crucial to the usefulness of telehealth to their treatment with time lags and other disruptions immediately impacting on their sense of confidence in the care delivered. Some noted that they needed to revert to the phone when technical problems were experienced by themselves or their provider.

C2: “*We had lots of tech difficulties that day…sometimes if the bandwidths don't match up there's then an issue with the transition…the timing of people talking and so forth…at times we couldn't understand his questions and we were a bit unsure what to reply…*.”

C3: “*It was mainly with the internet and who was having issues with the internet at the time…and we just ended up going to phone calls if it was dropping out too much*.”

#### Recommendations for future telehealth use

Consumers generally endorsed availability of telehealth as part of a blended approach to mental health service provision beyond the pandemic. Even when expressing a preference for face-to-face services over telehealth, they agreed that having telehealth as an option for consumers alongside face-to-face mental health services was important to support access and provide choice. Some consumers acknowledged that face-to-face services were important for them to address isolation and build their confidence in daily living skills such as negotiating social encounters but emphasised that telehealth could help them continue with treatment when facing barriers to attending sessions face-to-face and was therefore an important piece in their recovery.

C3: “*It's a good thing that came out of COVID, a silver lining…seeing that this has worked and it's helped people's mental health. I think just stopping it and not having it as an option will be detrimental for some people and so I'd be disappointed if it was completely stopped*.”

C5: “*I’d like to see it as a permanent thing…for people that struggle to get into appointments…if it was a 35-degree day, or if it was pouring rain, it would make it so hard. I think it would be good to have that (as an) ongoing option*.”

C6: “*If I had the options and everything was in a safe environment [with COVID], I would do face-to-face because there’s more of a connection thing and I guess when you are in person with someone who is treating you, you’ve got more of a connection and more of an understanding*.”

#### Service leaders and managers

Given the potential for individual views and perspectives in this small and specific stakeholder group to be identifiable, qualitative data was analysed with a view to identifying high level service factors relevant to future implementation of telehealth in similar services. These were collated and summarised in Table 2. Key enablers and barriers to effective establishment of the telehealth service were consistent with service providers. Enablers included access to infrastructure and space, prior experience and skills for staff, and strong existing relationships between the health service and the tertiary institution, which facilitated common goals and a shared commitment to the local community. Barriers included infrastructure gaps, early negative experiences and discouragement, some inertia of mode (attributed to established practice), and insufficient leadership and advocacy for telehealth, which left it easy to choose more familiar modes of contact. Consistent with providers, implementation factors were noted to vary with both consumer presentation and clinician profession, with more medicalised and therapeutic service delivery, e.g., psychiatric medication reviews, CBT, easier to adapt to telehealth. By contrast, psychiatric nursing and social work services reported seeing telehealth as unnecessary, inconsistent with their hands-on modes of practice, and unsuitable for complex psychosocial consumer presentations, particularly associated with paranoia and/or significant trust issues. Additionally, concerns were raised about consumer access to suitable phones and home internet to use these services, particularly for acute presentations and low socioeconomic status consumers.

**Table 2:** Summary of qualitative interview findings

	Consumers	Providers	Service leaders and managers
Enablers	Facilitates rapport similar to face to face	Type of clinical work (physical assessment vs. extended mental health assessment and psychological therapy)	Adequate infrastructure (computer, internet) and space
		Early success was motivating	Prior experience
Barriers	Unreliable and inconsistent internet connectivity	Inadequate infrastructure (hardware, internet access and speed)	Lack of peripherals
	Clinical information and cues may be lost	Lack of appropriate space (privacy)	Suitable space for privacy
	Platform hard to navigate	Cumbersome platform	Early negative experiences
	Distrust of technology	Lack of confidence with technology	Lack of leadership
	Fear of intrusion into private space		
	Discomfort with video images of self		
Recommendations for future use	Should be available as an option to provide access and choice	Investment in robust, stable, user-friendly platform and infrastructure	Provision of in-person training
		Development of guidelines for use	Identify clinician and consumer champions
		Provide/develop strong leadership	Schedule staff rotations into telehealth
		Provide mechanism to share knowledge and experience	

Recommendations for future telehealth options included retaining telehealth within a flexible suite of service modalities, with particular utility for those in remote settings, quick and more frequent reviews, and to increase the safety and efficiency of service delivery. To address identified barriers, stakeholders felt that more structured support, including in-person training and champions for both providers and consumers, and scheduling staff into rotating sessions to build confidence, would be valuable.

## Discussion

This mixed methods evaluation shows that telehealth was an important component of service delivery in a public mental health provider during the initial response to COVID-19 in early 2020 when face-to-face contacts decreased by 62%. Telephone sessions were the primary method for filling this service gap, and videoconferencing increased albeit to a lesser extent.

Telehealth has been shown to be an important tool to maintain delivery of health care services during the COVID-19 pandemic while keeping consumers and providers safe [17]. While COVID-19 presented challenges for providers and consumers to deliver and receive usual care because of social distancing measures and lockdowns, it also catalysed uptake of telehealth services [18]. Previous economic evaluations have also found that telehealth used for mental health consultations were cost-effective and possibly cost saving [4, 5]. This suggests that telehealth would be a sustainable alternative to in-person visits for improving access and consumer choice in mental health systems.

Our findings of more frequent use of telephone over videoconferencing were comparable to data from Federally Qualified Health Centers in the United States, that showed 63% of behavioural health visits in March 2020 occurred by telephone while 13.9% were conducted over video telehealth [19]. However, they contrast with reported telehealth use within private mental health providers across Australia during the pandemic. Analysis of Australian Medicare data showed greater use of video conferencing to provide focussed psychological strategies under the Better Access initiative and additional COVID support items compared to telephone contacts during March–April 2020 noting the highest uptake of video conference items in Victoria potentially related to better digital infrastructure compared to other states [20].

We found that the type of telehealth contacts (videoconference or telephone) varied across specific discipline groups within the mental health services. Qualitative interviews with providers suggested these differences were attributable to the types of clinical tasks required, such as physical examination and medication administration, and professional norms. This is consistent with telehealth uptake being related to the model of care, notably the acceptability of psychological therapies due to their ongoing nature. Additionally, some client groups were noted as more/less suitable for telehealth. Specifically, clients with limited technical literacy, and those with high anxiety and/or paranoia around using technology, were poorly suited to telehealth. In contrast, those requiring more regular review and maintenance treatment or focused psychological therapy, rather than opportunistic engagement, were felt to be more suited for telehealth. Additionally, young and more educated client groups, and clients with caring responsibilities and/or geographic distance, found telehealth offered advantages over face-to-face care. Telehealth was also noted to promote greater equity of access to specialist services, which is a critical issue for regional service delivery. This suggests that telehealth is a useful option but requires careful consideration to ensure it is used for appropriate tasks and client groups. Tertiary public mental health services have responsibility for the treatment of severe mental health populations who through a range of factors including homelessness and chronic symptoms may struggle to access technologies and connect through this medium. Other mental health services have noted similar concerns [21–23].

Generally successful implementation of telehealth in this study was evidenced through over 80% of consumers rating videoconferencing as good or excellent. This is comparable to the approximately 82% of participants rating their overall experience of telepsychiatry as good or excellent in a similar survey across 11 US states [24]. Nevertheless, further improvements are possible by focusing on both barriers and challenges reported by providers and consumers. Specific barriers included the sense of fatigue from continuous online engagement, and practical delays in the exchange of information, which required flexibility and adjustment for both clinicians and clients. Such barriers highlight the previously identified need for simpler telehealth systems which still maintain privacy, more stable and suitable digital hardware and internet connectivity for staff, and strategies to support clients in accessing telehealth [25, 26]. Additionally, the role of service guidelines to inform decision making, and identifying telehealth champions and mentors could support future implementation within a suite of clinical service offerings, consistent with prior evaluations of telehealth service development more broadly [27]. These results reflect similar findings in Australia and internationally [28–30] that have reported variable implementation of telehealth during the COVID-19 pandemic and recommended further exploration of best practice for its use in mental health services.

The study was designed to gather and understand consumer and provider perspectives on their access and use of telehealth services, its utility and quality, and with a view to enhancing telehealth for emergency and routine use in mental health care. Both consumers and providers in our study had favourable views of telehealth as essential and valuable as a routine component of future mental health care strategy, with similar findings reported by Thomas et al. [31], who recommended improving digital ecosystems and integrating telehealth into routine care. Barriers identified by clinicians highlight the need for investment in digital infrastructure in public mental health services which lag behind Medicare funded practices. Otherwise, in an on-line world, scaffolding of learning and adoption of technologies to enhance clinical care will be limited. Facilitators and barriers identified by consumers and providers in our study were similar to a review and clinical observations made by Chen et al. [32] from a US perspective and qualitative interviews undertaken by Liberati et al. [33] in England. One exception was that consumers in our study noted distrust of technology and fear of intrusion into private spaces. Chen et al. [32] noted a loss of privacy due to visibility of home environment as a risk to providers. Some conditions or person specific factors such as physical status for people with eating disorders emerged even at this very early stage of telehealth adoption. Further study is required to identify under what circumstances adequate translation of service versus risk of missing risk factors or potentially reinforcing unhelpful behaviours.

This mixed-methods evaluation leveraged rapid scale-up of telehealth services in a regionally based Area Mental Health service in response to the COVID-19 epidemic and ensuing social distancing and lockdown measures. One strength was the use of detailed administrative data to understand patterns of service use during the initial stage of the pandemic in comparison to a historical control. Qualitative interviews with over thirty service providers offered detailed insights into perceived barriers and facilitators during the rapid transition to telehealth. However, results from the online surveys were limited by the low response rates for both providers and consumers. Consumers completing surveys were mostly repeat users of videoconferencing and therefore more likely to be satisfied with the medium. Additionally, with only six consumer interviews undertaken, it is unlikely that all possible viewpoints were captured. We note that all consumers participating in the qualitative interviews were users of telehealth services and hence client barriers are likely underrepresented. The reported barriers and facilitators found in this research also need to be considered against the COVID-19 context and ensuing rapid transition to telehealth to extend and maintain mental health care. Deliberate deployment of an implementation framework such as the nonadoption, abandonment, scale-up, spread and sustainability (NASS) framework [34], commonly applied in routine technology innovations in healthcare, may have yielded better results.

## Conclusions

The rapid implementation of telehealth in mental health care due to COVID-19 provided an opportunity for evaluation through a natural experiment. Our findings highlight the utility of video conferencing telehealth as one option within a public mental health service supporting equity of access and consumer choice. Investment in technology, guidelines for appropriate use, and structured support are recommended to ensure telehealth is used to its full potential in the future.

## Supplementary Information

**Additional file 1: Table S1.** COVID Time line. **S2.** Consumer telehealth survey. **S3.** Provider telehealth survey. **S4.** Semi-structured interview outline – Consumers (MHDAS). **S5.** Semi-structured interview outline – Service providers (MHDAS). **Figure S6.** Videoconference utilisation trend over time from 2019 to 2020. **Figure S7.** Telephone utilisation trend over time from 2019 to 2020. **Table S8.** Age distribution of consumers from service use data in 2019 and 2020. **Table S9.** Contact type by service subcentre comparing 2019 to 2020. **Table S10.** Characteristics of consumers providing survey responses. **Table S11.** Service of the clinicians providing survey responses.

## Data Availability

Some data generated and/or analysed during the current study may be made available upon request from the corresponding author subject to ethics requirements.

## Abbreviations

COVID-19: Coronavirus disease 2019
SARS: Severe acute respiratory syndrome
MERS: Middle eastern respiratory syndrome
WHO: World Health Organization
