# Supplementary material for: Telehealth service delivery in an Australian regional mental health service during COVID-19: a mixed methods analysis
Source: Int J Ment Health Syst. 2022 Aug 19;16:43. doi: 10.1186/s13033-022-00553-8 (PMC9388972; doi:10.1186/s13033-022-00553-8)
Supplement: Supplementary file 1 — Additional file 1: Table S1. COVID Time line. S2. Consumer telehealth survey. S3. Provider telehealth survey. S4. Semi-structured interview outline – Consumers (MHDAS). S5. Semi-structured interview outline – Service providers (MHDAS). Figure S6. Videoconference utilisation trend over time from 2019 to 2020. Figure S7. Telephone utilisation trend over time from 2019 to 2020. Table S8. Age distribution of consumers from service use data in 2019 and 2020. Table S9. Contact type by service subcentre comparing 2019 to 2020. Table S10. Characteristics of consumers providing survey responses. Table S11. Service of the clinicians providing survey responses. [file 13033_2022_553_MOESM1_ESM.docx]

# **Additional file 1**

**Table S1. COVID Time line**

| **State and federal directions** |  | **Local Barwon Health & Mental Health Drug and Alcohol Services directions** |
| --- | --- | --- |
| Australian Health Sector Emergency Response Plan for novel corona virus | 27 Feb |  |
|  | 12 March | Meetings on-line, consumer screening, stay home if unwell (known case in Geelong) |
| State of emergency Victoria | 16 March | Staff advised to register for Health Direct |
|  | 19 March | Service level response plans finalised |
|  | 20 March | Maintain PPE stock low levels (acute priority), can see in person but look for alternatives |
| Stage 1 restrictions Victoria: Social distancing & closure of nonessential services | 22 March |  |
|  | 23 March | Face to face tribunal hearings suspended & moved to telehealth |
| Stage 2 restrictions Victoria | 24 March | Psychology clinic directed to work from home |
| National COVID coordination commission | 25 March | Script released re: no face to face services, ongoing care via phone/telehealth |
| Stage 3 restrictions | 30 March | Go live Central sites and phone tree alterations |
|  | 12 April | No new cases Geelong |
|  | 14 April | Agreement for remote access for trainees |
|  | 16 April | Deakin clinic open |
|  | 24 April | Deakin trainee telehealth orientation |
| Easing of restrictions (visitors to house, outside activities) | 18 May |  |
|  | 21 May | Draft restoration plan |
|  | 1 June | Service restoration phase 2 (staff) |
|  | 13 June | Service restoration phase 3 (consumers) |
| Household restrictions | 22 June |  |
|  | 25 June | Social distancing update (staff level vs consumers) |
| 10 Postcode lockdown | 30 June |  |
| Interstate boarder closure VIC-NSW | 8 July |  |
| Melbourne Mitchell lockdown | 9 July |  |
|  | 17 July | No Melbourne-Geelong transfers, Masks in public areas |
|  | 20 July | Colac outbreak |
| Melbourne face masks | 22 July |  |
| State of disaster Victoria; Regional Vic stage 3 restrictions (face masks), Melb stage 4 (curfew) | 2 Aug |  |
|  | 7 Aug | Masks at all times, eye wear for face to face |
|  | 10 Aug | Additional Deakin offices configured |
|  | 14 Aug | Trainee restrictions Melbourne-Geelong |
|  | 27 Aug | Noticeable breakdown in Barwon Health IT |

**S2. Consumer telehealth survey**

| 1. **Your Age** |  |  | 1. **Your Gender** |  |
| --- | --- | --- | --- | --- |
| 18 - 29 |  |  | Female |  |
| 30 - 44 |  |  | Male |  |
| 45 - 64 |  |  | Other |  |
| 65 - 74 |  |  | Prefer not to say |  |
| 75 - 84 |  |  |  |  |
| 85+ |  |  |  |  |

1. **How satisfied were you with the following:**

|  |  | Poor |  | Fair |  | Good |  | Excellent |
| --- | --- | --- | --- | --- | --- | --- | --- | --- |
| The quality of the technical connection (i.e. image and/or sound) |  |  |  |  |  |  |  |  |
| Your personal comfort in using telehealth |  |  |  |  |  |  |  |  |
| Your overall treatment experience at telehealth |  |  |  |  |  |  |  |  |

1. Would you use Telehealth again? Yes N o

**COMMENT: If you'd like to provide any other comments, please include here.**

______________________________________________________________________

**S3. Provider telehealth survey**

1. How would you rate the technical quality of this telehealth consultation?

1. Excellent
2. Good
3. Fair
4. Poor

2. Were you able to achieve your assessment/treatment goals with this consumer in this telehealth session?

1. Yes
2. No
3. Can’t be determined / unable to say

**S4. Semi-structured interview outline – Consumers (MHDAS)**

**Describe purpose of interview and overall project – aims, methods, follow up**

**Reiterate informed consent, length and purpose of the interview**

*As described in the Plain Language Statement , the interview component of the study aims to understand your experiences of telehealth within the Barwon Health Mental Health Drugs and Alcohol Services (MHDAS).* It aims to understand the role that telehealth has had in supporting the consumers of MHDAS during Covid 19 as well as whether it might be helpful for consumers to engage with the service into the future [define telehealth]*. As described in the PLS, the interview will be audio-recorded. This helps us transcribe all interviews. All data will remain confidential. Your identity will remain anonymous in any publications that come out of the research study. All data collected will be destroyed after 7 years as per ethics requirements. Do you consent to participate in the interview?*

**Briefly outline interview structure**

*I will be asking you several questions related to your attitudes towards and experiences of telehealth as part of your care with Barwon Health MHDAS. Depending on your responses, I may ask follow up questions to assist developing a comprehensive understanding of your thoughts and decisions. If there is anything you do not wish to answer, please just tell me and we can move on to the next question. The interview is expected to be of at around 45 minutes in duration – or however long you are happy to discuss. Do you have a time that you need to be finished by?*

*Do you have any questions?*

**Questions for consumers:**

1. **Did you had experience of telehealth as a means to receive health services (including mental health or drug and alcohol services) in the past?  If so, what were the benefits and drawbacks when comparing this experience to face to face health care.**

*(This is a general prompt to understand any previous experience of telehealth)*

1. **Thinking about your experience of telehealth with MHDAS, what factors influenced your decision to receive your care in this way? Were there other ways of receiving care that were offered or that you required during the COVID 19 period?**

1. **Did you have any concerns about engaging in telehealth? If so, how did you go about having these concerns addressed?**

1. **Thinking about these experiences of telehealth in your care with MHDAS, were there ways that made it easier to attend and/or participate? Were there aspects of telehealth that made it more difficult to continue your care with MHDAS or participating more difficult?**

1. **Thinking about your experiences of telehealth to receive care with MHDAS, in what ways was it similar to your usual way of receiving care? In what ways was it different?**

1. **How effective did you find telehealth as a way to meet the goals you had for receiving care at MHDAS?**

1. **If telehealth was an option for you to receive care from MHDAS in the future, would you want to continue to receive care in this way?**

1. **Do you have any recommendations that would believe would enhance the help that you might receive telehealth in the future?**

1. **Is there anything else that you would like to share about your experiences with telehealth with MHDAS?**

*Thank-you for your time*

**S5. Semi-structured interview outline – Service providers (MHDAS)**

**Describe purpose of interview and overall project – aims, methods, follow up**

**Reiterate informed consent, length and purpose of the interview**

*As described in the Plain Language Statement, the interview component of the study aims to understand the feasibility and acceptability of telehealth as service provision option for consumers of the*service. It aims to understand the role of telehealth in the context of the current pandemic as well as it’s potential utility for the service into the future [define telehealth]*. As described in the PLS, the interview will be audio-recorded. This helps us transcribe all interviews. All data will remain confidential. Your identity will remain anonymous in any publications that come out of the research study. All data collected will be destroyed after 7 years as per ethics requirements. Do you consent to participate in the interview?*

**Briefly outline interview structure**

*I will be asking you several questions related to your attitudes towards and experiences of telehealth as a mode for delivering mental health and drugs and alcohol services to consumers of our service and your perception of its effectiveness, and feasibility within a mental health service. Depending on your responses, I may ask follow up questions to assist developing a comprehensive understanding of your thoughts and decisions. If there is anything you do not wish to answer, please just tell me and we can move on to the next question. The interview is expected to be of at around 45 minutes in duration – or however long you are happy to discuss. Do you have a time that you need to be finished by?*

*Do you have any questions?*

**Questions for service providers:**

1. **Have you had experience of using telehealth as a mode to deliver mental health services in the past?**

*(This is a general prompt to understand any previous experience of telehealth)*

1. **Can you briefly tell me about your utilisation of telehealth as a service provision model with your clients during the past 3 months**

**If providers have used the service model:**

1. **(a)** **Thinking about your incentives or motivations to utilise telehealth in your work with consumers, what factors were important in making your decision?**

**(b)** **What was the main reason you chose telehealth to work with consumers? If there were other service options (e.g. phone calls, face to face assessments), what was the main motivator for you to adopt telehealth?**

**(c)** **Thinking of your experience of using telehealth in your work, how was it similar to your usual way of working? In what ways was it different?**

**(d)** **Thinking about your experiences of using telehealth in your work, what (if any) aspects of the modality made it easy to use? What (if any) aspects of the modality made it difficult to use?**

**(e)** **Can you talk briefly about how the telehealth service approach was received by consumers that you worked with. Were there consumer groups that this mode was particularly effective for? Were there consumer groups that this mode was not effective for? Why?**

**(f)** **If there was an opportunity to incorporate telehealth into your work with consumers as part of a usual model of care, would you utilise it and why? If so, in what circumstances would you have a preference for using it?**

**(g)** **What factors do you think would influence the success of incorporating telehealth as part of a model of care in the service ?**

1. **Is there anything else that you would like to share about your experiences with telehealth over the past 2-3 months**

**If providers have not used telehealth:**

**5.** **(a)** **Considering that you haven’t taken up telehealth as a service mode in your work with consumers, what influenced your decision?**

**(b)** **Were there any specific barriers that you can identify that influenced this outcome for you in your work ?  Do you have ideas about how these barriers might be overcome?**

**(c)** **Are there circumstances where you foresee telehealth would be a useful service mode for your consumers?**

**Any further questions that seem relevant given responses to all the above.**

*Thank-you for your time*

**Figure S6. Videoconference utilisation trend over time from 2019 to 2020**


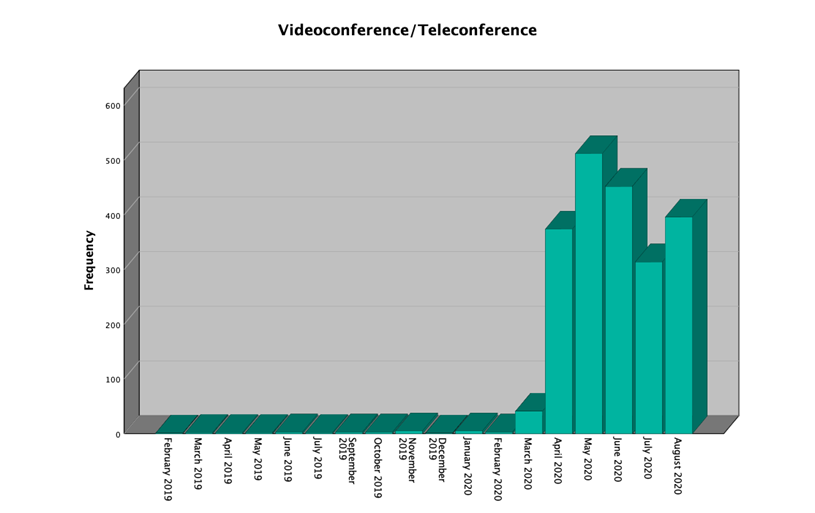


**Figure S7. Telephone utilisation trend over time from 2019 to 2020**


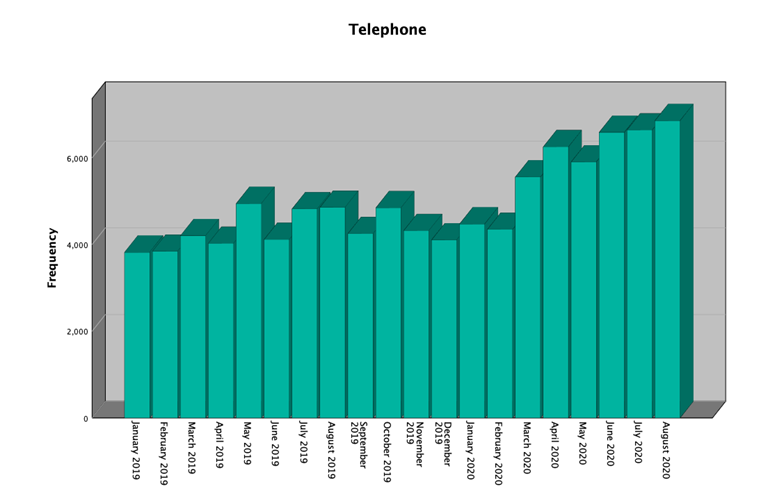


**Table S8. Age distribution of consumers from service use data in 2019 and 2020**

| **Age group (years)** | **April - May 2019** | **April - May 2020** |
| --- | --- | --- |
|  | **n (%)** | **n (%)** |
| 0-4 | 9 (0.04) | 0 (0.00) |
| 5-9 | 82 (0.41) | 70 (0.40) |
| 10-14 | 418 (2.09) | 415 (2.39) |
| 15-19 | 1,045 (5.22) | 1,098 (6.32) |
| 20-24 | 1,807 (9.02) | 1,576 (9.08) |
| 25-29 | 1,415 (7.07) | 1,287 (7.41) |
| 30-34 | 1,532 (7.65) | 1,187 (6.84) |
| 35-39 | 1,540 (7.69) | 1,289 (7.43) |
| 40-44 | 1,436 (7.17) | 1,276 (7.35) |
| 45-49 | 1,581 (7.90) | 1,819 (10.48) |
| 50-54 | 1,054 (5.60) | 980 (5.65) |
| 55-59 | 819 (4.09) | 694 (4.00) |
| 60-64 | 603 (3.01) | 661 (3.81) |
| 65-69 | 744 (3.72) | 461 (2.66) |
| 70-74 | 350 (1.75) | 503 (2.90) |
| 75-79 | 237 (1.18) | 311 (1.79) |
| 80-84 | 131 (0.65) | 179 (1.03) |
| 85-89 | 29 (0.14) | 32 (0.18) |
| 90-94 | 68 (0.34) | 69 (0.40) |
| 95-99 | 0 (0.00) | 23 (0.13) |
| Unknown | 5,123 (25.59) | 3,430 (19.76) |
| Total | 20,023 (99.99) | 17,360 (100.01) |

**Table S9. Contact type by service subcentre comparing 2019 to 2020**

|  | **April - May 2019** | |  |  |  | **April - May 2020** | |  |  |  | **Percent change 2019 to 2020** | | |
| --- | --- | --- | --- | --- | --- | --- | --- | --- | --- | --- | --- | --- | --- |
| **Subcentre** | **Face-to-face** | **Other syncronus** | **Telephone** | **VC** | **Total** | **Face-to-face** | **Other syncronus** | **Telephone** | **VC** | **Total** | **Face-to-face** | **Telephone** | **VC** |
| **Acute Unit -Aged Persons** | - | - | - | - | - | 2 | 0 | 9 | 0 | 11 |  |  |  |
| **Aged Psychiatric Service** | 798 | 7 | 876 | 0 | 1,681 | 261 | 14 | 1127 | 166 | 1568 | -67% | 29% | 100% |
| **Childrens MHS** | 655 | 0 | 779 | 2 | 1,436 | 36 | 2 | 895 | 246 | 1179 | -95% | 15% | 12200% |
| **Families where a Parent - FaPMI** | 73 | 0 | 48 | 0 | 121 | 3 | 2 | 43 | 0 | 48 | -96% | -10% | 0% |
| **Child & Youth Triage** | 2 | 0 | 776 | 0 | 778 | 0 | 15 | 630 | 0 | 645 | -100% | -19% | 0% |
| **Forensic** | 299 | 5 | 19 | 0 | 323 | 18 | 0 | 76 | 36 | 130 | -94% | 300% | 100% |
| **MAHRS Geelong Magistrates Court** | - | - | - | - | - | 60 | 19 | 14 | 2 | 95 |  |  |  |
| **Access & Consultation/Liaison** | 1,132 | 69 | 1,441 | 0 | 2,642 | 1011 | 17 | 1743 | 2 | 2773 | -11% | 21% | 100% |
| **Court Liaison** | 2 | 0 | 0 | 0 | 2 | - | - | - | - | - |  |  |  |
| **JIGSAW** | 1,399 | 0 | 1,498 | 1 | 2,898 | 138 | 35 | 2054 | 155 | 2382 | -90% | 37% | 15400% |
| **Combined CMHT** | 5,443 | 16 | 3152 | 0 | 8,611 | 2578 | 23 | 4824 | 42 | 7467 | -53% | 53% | 100% |
| **Primary Mental Health Partners** | 311 | 0 | 41 | 0 | 352 | 29 | 0 | 97 | 80 | 206 | -91% | 137% | 100% |
| **Clozapine** | 42 | 0 | 96 | 0 | 138 | 0 | 1 | 155 | 0 | 156 | -100% | 61% | 0% |
| **Perinatal Emotional Health Program** | 41 | 0 | 70 | 0 | 111 | 16 | 3 | 172 | 3 | 194 | -61% | 146% | 100% |
| **Adult Intensive Complex Care Packages** | 195 | 0 | 32 | 0 | 227 | 1 | 0 | 1 | 0 | 2 | -99% | -97% | 0% |
| **Homeless Outreach** | 114 | 4 | 40 | 0 | 158 | 0 | 0 | 12 | 0 | 12 | -100% | -70% | 0% |
| **Eating Disorders Service** | 300 | 0 | 99 | 1 | 400 | 8 | 2 | 302 | 154 | 466 | -97% | 205% | 15300% |
| **CASEA** | 143 | 0 | 2 | 0 | 145 | - | - | - | - | - |  |  |  |
| **Combined PACER** | - | - | - | - | - | 24 | 0 | 2 | 0 | 26 |  |  |  |
| **Total** | 10,949 | 101 | 8,969 | 4 | 20,023 | 4185 | 133 | 12156 | 886 | 17360 | -62% | 36% | 22050% |

**Table S10. Characteristics of consumers providing survey responses**

| **Characteristic** | **n (%)** |
| --- | --- |
| **Age range** |  |
| 16-17 years | 1 (4) |
| 18-29 years | 6 (24) |
| 30-44 years | 2 (8) |
| 45-64 years | 13 (52) |
| 65-74 years | 3 (12) |
| **Sex** |  |
| Female | 23 (88) |
| Male | 3 (12) |
| **First videoconference telehealth session** |  |
| Yes | 1 (4) |
| No | 25 (96) |
| **Service used** |  |
| Adult | 8 (31) |
| Young persons (Jigsaw) | 1 (4) |
| Child and adolescent | 4 (15) |
| Drug and alcohol | 3 (11) |
| Eating disorders | 1 (4) |
| Therapy team | 9 (35) |

**Table S11. Service of the clinicians providing survey responses**

| **Service** | **n (%)** |
| --- | --- |
| Adult | 8 (9) |
| Young persons (JIGSAW) | 6 (7) |
| Child and adolescent | 22 (25) |
| Therapy team | 52 (59) |
